# Alcohol and tea consumption are associated with asymptomatic erosive esophagitis in Taiwanese men

**DOI:** 10.1371/journal.pone.0173230

**Published:** 2017-03-06

**Authors:** Chung-Hsin Chang, Cheng-Pin Wu, Jung-Der Wang, Shou-Wu Lee, Chi-Sen Chang, Hong-Zen Yeh, Chung-Wang Ko, Han-Chung Lien

**Affiliations:** 1 Division of Gastroenterology, Taichung Veterans General Hospital, Taichung, Taiwan; 2 Health Examination Center, China Medical University Hospital, Taichung, Taiwan; 3 Preventive Medicine Center, China Medical University Hospital, Taichung, Taiwan; 4 Department of Public Health, College of Medicine, National Cheng Kung University, Tainan, Taiwan; 5 Department of Internal Medicine, Chung Shan Medical University, Taichung, Taiwan; 6 Department of Internal Medicine, National Yang-Ming University, Taipei, Taiwan; University Hospital Llandough, UNITED KINGDOM

## Abstract

**Objective:**

Asymptomatic erosive esophagitis (AEE) is commonly found in men, and might be a risk factor of developing esophageal adenocarcinoma. We aimed to determine if specific dietary habits increase the risk of AEE in asymptomatic Taiwanese men.

**Methods:**

We recruited male adults undergoing upper gastrointestinal endoscopy for health check. We excluded subjects with reflux symptoms, or taking anti-reflux medications or drugs that potentially impair lower esophageal sphincter function or cause mucosal injury. The frequency of consuming reflux-provoking diets including alcohol, tea, coffee, tomato/citric juice, chocolate, sweet food, and spicy food was assessed. The erosive esophagitis was diagnosed based on the Los Angeles Classification after endoscopy. Frequent consumption of a specific diet was defined as ≥4 days/week of consuming that diet.

**Results:**

A total of 1256 participants were recruited. After excluding 424 ineligible subjects, AEE was identified in 180 (22%) among 832 asymptomatic subjects. The risk of AEE increased with the number of days per week of consuming alcohol or tea: nondrinkers (19%, 17%), occasional drinkers (<1 day/week; 19%, 15%), regular drinkers (1–3 days/week; 26%, 21%), frequent drinkers (4–6 days/week; 32%, 22%), and daily drinkers (42%, 28%), respectively (trend test P < 0.001 for both). Multivariate analysis showed that hiatus hernia (adjusted odds ratio (aOR) 5.0, 95% confidence interval (CI) 2.6–9.6), drinking alcohol ≥4 days/week (aOR 2.3, 95% CI 1.3–4.0), and drinking tea ≥4 days/week (aOR 1.6, 95% CI 1.1–2.3) are independent risk factors of AEE. The risk of AEE was 3.8 times greater for those drinking both alcohol and tea ≥4 days/week than the non-drinkers.

**Conclusions:**

Frequent alcohol and tea consumption increased the risk of AEE in Taiwanese men.

## Introduction

The prevalence of erosive esophagitis (EE), the most common manifestation of esophageal injury of gastroesophageal reflux disease (GERD) [1], has increased throughout Asia over the past decade [2]. In Taiwan, it was estimated to range from 9.0% to 24.6% among adults who underwent a screening endoscopy during a health check [3]. These rates are within the range of those reported in general population studies conducted in Sweden (15.5%, Kalixanda) [4] and Italy (11.8%, Loiano-Monghidoro) [5]. Several health-check studies from Asia including Taiwan showed that 11.6% to 45.3% of subjects with EE had no reflux symptoms, i.e., asymptomatic erosive esophagitis (AEE), particularly in men [6–9], which was also in line with the Kalixanda and the Loiano-Monghidoro studies, showing up to 36.8% and 32.8% of patients with EE were asymptomatic, respectively [4,5]. Although the natural history and the significance of AEE remain unknown, it is important to note that EE is a major risk factor for developing Barrett’s esophagus (BE) [10], which is the precursor of esophageal adenocarcinoma (EAC) caused by chronic GERD, and was detected in 25% of asymptomatic men older than 50 years of age undergoing screening sigmoidoscopy for colorectal cancer [11]. Therefore, subjects with AEE may theoretically be at risk of developing EAC, as 40% of patients who develop EAC have never experienced any symptoms of reflux and are therefore less likely to seek medical attention [12].

Certain foods or beverages, such as chocolate or alcohol may provoke GERD. Therefore, dietary modifications are often used as a first-line treatment for subjects with GERD [13]. However, the current guidelines do not support dietary restrictions as being an effective therapy for GERD [14], because of a lack of evidence to support that either frequent consumption of certain foods cause the disease, or that an avoidance of specific diets reduces the disease’s occurrence. In fact, it is generally difficult to evaluate the cause and effect between dietary habits and GERD, because patients with reflux symptoms may tend to avoid certain foods that would provoke their symptoms [15]. In this regard, subjects with AEE may be considered the preferred study population when evaluating the relationship between dietary habits and GERD.

This study aimed to determine whether frequent consumption of potential reflux-provoking food and beverages such as alcohol, tea, coffee, tomato/citric juices, chocolate, sweet food, and spicy food [14] may be associated with the risk of AEE in Taiwanese men who were undergoing a screening upper endoscopy during a health check.

## Materials and methods

### Participants

The study population was 1256 male subjects who underwent self-paid health checks in Taichung Veterans General Hospital from March 2002 to September 2002. After obtaining their informed consent, each participant underwent an interview to discuss their medical history, along with a physical examination, an esophagogastroduodenoscopy (EGD), multiphasic blood screening, a chest radiograph, an abdominal ultrasound, and a colonoscopy. All participants were over 20 years of age, and most of them were middle-class citizen and were apparently healthy. We excluded subjects with upper gastrointestinal surgery or tumors, BE, any acute illness, refusal for EGD, refusal to participate in the study, or inability to verbally communicate. Subjects who were taking medications that commonly impair the lower esophageal sphincter (LES) such as aminophyllines, anticholinergics, beta-adrenergic agonists, nitroglycerins, and benzodiazepines; or cause direct mucosal injury such as bisphosphonates, ferrous sulfate, doxycycline, non-steroid anti-inflammatory drugs, and ascorbic acid, were also excluded. Participants who answered “yes” to any one of the following 3 questions were considered symptomatic and were also excluded: (1) “In the last 3 months, have you experienced any symptoms of heartburn at least once in a month? (The term “heartburn” was defined as a burning pain or discomfort behind the breast bone.)” [16] (2) “In the last 3 months, have you experienced any symptoms of acid regurgitation at least once in a month? (The term “acid regurgitation” was defined as a bitter or sour fluid rising to the throat or mouth.)” (3) “In the last 3 months, have you taken any anti-reflux medications to relieve your upper digestive tract from any symptoms such as upper abdominal, retrosternal, or throat discomfort, more than once in a month? (The anti-reflux medications may include antacids, proton pump inhibitors, anti-histamine 2 receptors blockers, or pro-motility agents).” The questionnaire was administered prior to the EGD exam.

The research protocol was first approved by the Institutional Review Board of Taichung Veterans General Hospital (TCVGH No: C06284) and follows the principles of the Declaration of Helsinki. All participants provided written informed consent prior to any procedures. The data and questionnaires were analyzed anonymously, so that subjects could not be personally identified in the data analysis.

### Questionnaire

A structured, self-administered questionnaire was developed to evaluate the dietary habits of Taiwanese and the potential reflux-provoking food and beverages commonly consumed by them. These include coffee, chocolate, alcohol, tea, tomato/citric juices, sweet food, and spicy food. The questionnaire contained 7 items, each asking the number of days per week one certain kind of food or beverage was consumed. For example, “Within the prior 3 months, how often on average did you drink alcohol per week? (at least a glass of wine or spirits, or a can of beer each time)”. Another estimated amount of beverage use was defined as at least one cup of coffee or tea, one bottle of tomato/citric juice each time, and one occasion for consuming chocolate, sweet foods, and spicy foods. Subjects were allowed to choose either one of the following answers: (1) never, (2) less than one day per week, (3) between one to three days per week, (4) between four to six days per week, or (5) almost daily or more frequently than daily.

Before the study, a pre-test of the questionnaire was conducted among 25 randomly selected hospital staff members to see whether or not they had any difficulty in understanding the questionnaire, which was then modified according to their suggestions. Subsequently, the test-retest reliability was conducted in the same group, by comparison of the two tests 7–14 days apart, and showed a median kappa statistic of 0.85 (interquartile range, 0.79–0.9). The questionnaire would usually be completed within one minute. The same study nurse confirmed the completeness of the questionnaire which had been filled out by the participants immediately before the EGD exam.

### Esophagogastroduodenoscopy

The standard practice in this program employed a group of experienced endoscopists (H-C,L; C-S,C; H-Z,Y; C-W,K) to perform EGDs in order to screen for early upper gastrointestinal malignancy. The endoscopists were unaware of each subject’s symptoms prior to the procedure, in order to avoid any information bias during the endoscopic assessment [17]. Each subject underwent an unsedated EGD. A standard electronic video gastroscope system (Olympus Inc., Tokyo, Japan) was used to assess EE, including the use of an Olympus GIF XQ-240 for the study, and static photographs were recorded on compact disks (CD-ROM). The severity of esophagitis was classified according to the Los Angeles classification (grades A-D) [18]. To evaluate the reliability of endoscopic diagnosis of EE, another experienced endoscopist (S-K,P) reexamined 80 endoscopic images randomly selected from study subjects. The inter-observer agreement between the performing endoscopist and the reviewing endoscopist was good to excellent with a kappa statistic of 0.8.

### Statistical analysis

To determine the effect of dietary habits on AEE, we measured the prevalence of AEE as determined through the frequency of each food or beverage consumption (never, <1 day/week, 1–3 days/week, 4–6 days/week, daily) and then evaluated the dose-response effect using the Cochran-Armitage trend test. All data, including demographic data (age, body mass index (BMI)), dietary habits (alcohol, tea, coffee, chocolate, tomato/citric juice, spicy and sweet food intake), and endoscopic findings, were compared between subjects with and without AEE. The Chi-square test was used for analyzing categorical data, and the Student’s t test was used for continuous data. BMI categories were defined as, normal weight (<23.9 kg/m^2^), overweight (24 to 26.9 kg/m^2^) and obesity (≥27 kg/m^2^), as based on Taiwan criteria [19]. Frequent food or beverage consumption was defined as ≥4 days per week for a specific food or beverage consumed in the last 3 months. Using unconditional logistic regression, we calculated the odds ratios (OR) as estimates of the relative risk and related 95% confidence intervals (CIs), in order to measure the association between frequent dietary consumption and AEE. Variables having a *p* <0.25 in the univariate analysis were entered as candidate risk factors in the multivariate logistic regression analysis, in order to identify independent risk factors of AEE [20]. All analyses were performed with SPSS 15.0 for Windows (Chicago, IL). A 2-sided probability value of less than 0.05 was considered statistically significant.

## Results

**Fig 1:**
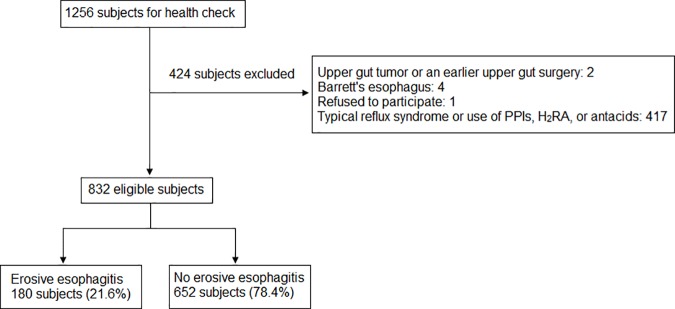
Enrollment of study subjects. (PPI, proton pump inhibitor; H_2_RA, histamine-2-receptor antagonist).

Of the 1256 men screened for EGD, 832 subjects were deemed eligible after excluding all subjects experiencing reflux symptoms, those taking anti-reflux medications or drugs that potentially impair LES function or cause mucosal injury (n = 417), those with a history of gastric cancer or previous upper gut surgery (n = 2), BE (n = 4), and any who refused to participate (n = 1), upon completion of a dietary habit evaluation (Fig 1). Among them, 21.6% (n = 180) were diagnosed with AEE and the vast majority (73.9%) of those with AEE were at a grade A severity level (Table 1). Dietary habits varied among the participants: 60.7% drank tea at least once weekly, while 39.2% drank tea almost daily (Table 1). Subjects found to have AEE were more likely to have a hiatus hernia (*p* < 0.0001), to drink alcohol (*p* = 0.003), and to drink tea (*p* = 0.008) than subjects without AEE (Table 1). There was no difference in age, BMI, or the frequency of consuming remaining foods or beverages between the two groups. Subjects with a history of reflux symptoms or those taking any anti-reflux medications appeared to have lower frequencies in the consumption of various food items which might provoke any symptoms.

**Table 1:** Demography, endoscopic findings, and dietary habits of asymptomatic male subjects with or without erosive esophagitis, and symptomatic male subjects.

	Subjects with reflux symptoms or those taking any medications that relate to erosive esophagitis* (n = 417)	Asymptomatic subjects		p**
		Erosive esophagitis (n = 180)	Normal controls (n = 652)	
**Demography**				
Age (years) (mean ± SD)	52.1 ± 12.6	51.8 ± 13.0	50.2 ± 12.0	0.1
Age ≥50 (years) (n (%))	221 (53.0)	97 (53.9)	328 (50.3)	0.4
BMI (kg/m^2^) (mean ± SD)	24.5 ± 3.0	24.6 ± 3.3	24.3 ± 3.3	0.2
BMI ≥24 (kg/m^2^) (n (%))	240 (57.6)	97 (53.9)	349 (53.5)	0.9
**Endoscopic findings** (n (%))				
Erosive esophagitis				
No erosive esophagitis	269 (64.5)	0 (0.0)	652 (100.0)	
Grade A	95 (22.7)	133 (73.9)	0 (0.0)	
Grade B	41 (9.8)	38 (21.1)	0 (0.0)	
Grade C	8 (1.9)	4 (2.2)	0 (0.0)	
Grade D	4 (1.0)	5 (2.8)	0 (0.0)	
Hiatus hernia	25 (6.0)	23 (12.8)	19 (2.9)	< 0.0001
Duodenal ulcer	77 (18.4)	22 (12.2)	98 (15.0)	0.4
Gastric ulcer	50 (11.9)	21 (11.7)	73 (11.2)	0.9
**Food or beverage habits** (n (%))				
Alcohol				
None	204 (48.9)	79 (43.9)	336 (51.5)	0.003
<1 day/week	91 (21.8)	40 (22.2)	173 (26.5)	
1–3 days/week	78 (18.7)	35 (19.4)	101 (15.5)	
4–6 days/week	20 (4.8)	8 (4.4)	17 (2.6)	
Everyday	24 (5.8)	18 (10.0)	25 (3.8)	
Tea				
None	85 (20.3)	25 (13.9)	123 (18.9)	0.008
<1 day/week	76 (18.2)	26 (14.4)	151 (23.2)	
1–3 days/week	62 (14.8)	26 (14.4)	95 (14.6)	
4–6 days/week	35 (8.4)	13 (7.2)	45 (6.9)	
Everyday	159 (38.1)	90 (50.0)	236 (36.2)	
Coffee				
None	223 (53.6)	95 (52.8)	339 (52.0)	0.2
<1 day/week	101 (24.2)	42 (23.3)	167 (25.6)	
1–3 days/week	54 (12.9)	10 (5.6)	64 (9.8)	
4–6 days/week	8 (1.9)	7 (3.9)	16 (2.5)	
Everyday	30 (7.2)	26 (14.4)	66 (10.1)	
Chocolate				
None	335 (80.3)	154 (85.6)	539 (82.7)	0.4
<1 day/week	67 (16.0)	26 (14.4)	103 (15.8)	
1–3 days/week	11 (2.6)	0 (0.0)	8 (1.2)	
4–6 days/week	3 (0.7)	0 (0.0)	0 (0.0)	
Everyday	1 (0.2)	0 (0.0)	2 (0.3)	
Tomato/citric juices				
None	151 (36.2)	81 (45.0)	239 (36.7)	0.1
<1 day/week	168 (40.2)	64 (35.6)	275 (42.2)	
1–3 days/week	79 (18.9)	29 (16.1)	101 (15.5)	
4–6 days/week	6 (1.4)	1 (0.6)	17 (2.6)	
Everyday	13 (3.1)	5 (2.8)	20 (3.1)	
Sweet food				
None	104 (24.9)	52 (28.9)	204 (31.3)	0.6
<1 day/week	158 (37.8)	68 (37.8)	268 (41.1)	
1–3 days/week	109 (26.1)	41 (22.8)	117 (17.9)	
4–6 days/week	20 (4.8)	3 (1.7)	13 (2.0)	
Everyday	26 (6.2)	15 (8.3)	49 (7.5)	
Spicy food				
None	202 (48.4)	89 (49.4)	354 (54.3)	0.6
<1 day/week	90 (21.5)	46 (25.6)	175 (26.8)	
1–3 days/week	83 (19.9)	30 (16.7)	82 (12.6)	
4–6 days/week	18 (4.3)	3 (1.7)	9 (1.4)	
Everyday	24 (5.8)	10 (5.6)	31 (4.8)	

BMI, body mass index; SD, standard deviation; n, number of subjects.

* Male subjects with symptoms of heartburn or acid regurgitation or those taking any anti-reflux medications, or drugs that potentially impair esophageal sphincter function or cause mucosal injury

** *p* value for comparison between asymptomatic male subjects with and without erosive esophagitis.

**Fig 2:**
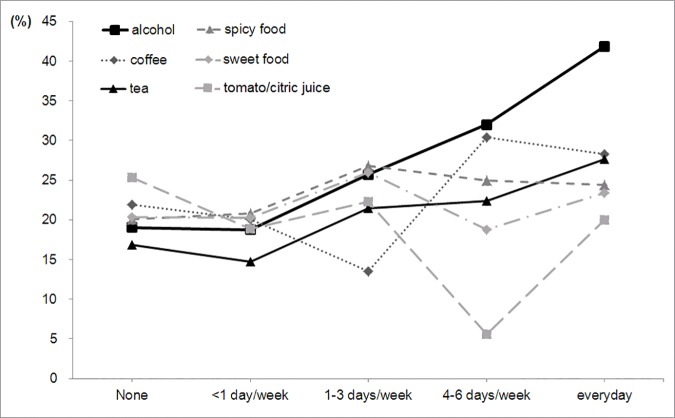
Proportions of asymptomatic erosive esophagitis by frequency of dietary habits in Taiwanese men. (trend test, *p* = 0.0003 for alcohol and *p* = 0.0005 for tea, respectively)

The prevalence of AEE increased in step with the increase in the number of days per week alcohol or tea was consumed (trend test, *p* = 0.0003 for alcohol and *p* = 0.0005 for tea, respectively), but not for other foods or beverages (Fig 2). The prevalence of AEE in participants drinking alcohol increased from 19.0% among non-drinkers, to 41.9% among daily drinkers (OR 3.1, 95% CI 1.6–5.9, *p* = 0.001); whereas with tea consumption, the AEE increased from 16.9% among non-drinkers, to 27.6% among daily drinkers (OR 1.9, 95% CI 1.1–3.1, *p* = 0.01; Fig 2).

In a multivariate logistic regression analysis model, a hiatal hernia (adjusted odds ratio (aOR) 5.0, 95% CI 2.6–9.6, *p* < 0.0001), drinking alcohol ≥4 days/week (aOR 2.3, 95% CI 1.3–4.0, *p* = 0.002), and drinking tea ≥4 days/week (aOR 1.6, 95% CI 1.1–2.3, *p* = 0.008) are all independently associated with AEE (Table 2). When compared with men who did not drink alcohol or tea, the estimated risk of AEE for men who drank both alcohol and tea ≥4 days/week, was 3.8 times greater (95% CI 1.7–8.7, Table 3).

**Table 2:** Crude and adjusted odds ratios and 95% confidence intervals of asymptomatic erosive esophagitis and associated factors in Taiwanese men.

	Univariate		Multivariate	
	OR (95% CI)	*p*	Adjusted OR (95% CI)	*p*
Hiatus hernia				
No	1.00 (reference)	<0.0001	1.00 (reference)	<0.0001
Yes	4.88 (2.59–9.19)		5.01 (2.62–9.56)	
Alcohol (days/week)				
<4	1.00 (reference)	0.001	1.00 (reference)	0.002
≥4	2.45 (1.46–4.13)		2.31 (1.34–3.99)	
Tea (days/week)				
<4	1.00 (reference)	0.001	1.00 (reference)	0.008
≥4	1.76 (1.26–2.45)		1.61 (1.13–2.28)	
Coffee (days/week)				
<4	1.00 (reference)	0.06	1.00 (reference)	0.2
≥4	1.56 (1.00–2.43)		1.35 (0.85–2.15)	
Tomato/citric juices (days/week)				
<4	1.00 (reference)	0.3	1.00 (reference)	0.1
≥4	0.57 (0.24–1.38)		0.47 (0.19–1.16)	

BMI, body mass index; OR, odds ratio; CI, confidence interval.

**Table 3:** Adjusted odds ratios and 95% confidence interval of asymptomatic erosive esophagitis by frequency of alcohol and tea consumption in Taiwanese men.

	Alcohol					
	None (n = 415)		<4 days/week (n = 349)		≥4 days/week (n = 68)	
	n/N (%)	Adjusted OR (95%CI)	n/N (%)	Adjusted OR (95%CI)	n/N (%)	Adjusted OR (95%CI)
Tea						
None (n = 148)	14/106 (13.2)	1.00 (reference)	7/33 (21.2)	1.8 (0.6–4.9)	4/9 (44.4)	5.7 (1.4–23.8)
<4 days/week (n = 298)	25/142 (17.6)	1.3 (0.6–2.7)	23/147 (15.6)	1.2 (0.6–2.5)	4/9 (44.4)	4.8 (1.1–20.8)
≥4 days/week (n = 384)	40/166 (24.1)	1.8 (0.9–3.5)	45/169 (26.6)	2.5 (1.3–4.9)	18/49 (36.7)	3.8 (1.7–8.7)

n, patients with erosive esophagitis; OR, odds ratio; CI, confidence interval. ORs were adjusted for hiatus hernia. Missing data of tea drinking in one of 415 subjects who did not drink alcohol and in one of 68 subjects who consumed alcohol ≥4 days/week.

## Discussion

This study explored the prevalent rates and risk factors of AEE among asymptomatic Taiwanese men, focusing on their dietary habits. Although we discovered that frequent consumption of alcohol and/or tea were independently associated with increased risks of AEE, with an apparent dose-response relationship, it does not necessarily imply that they are causally related. However, we make the following arguments to support such a hypothesis: First, we have deliberately included participants without any previous history of reflux symptoms, or those taking any medications that may be related to reflux or esophageal mucosal injury. Thus, the association cannot be explained by any possible coexistence of symptomatic GERD or medications. Secondly, because we have constructed a multivariable, logistic regression model by including hiatal hernia, coffee, chocolate, and other common dietary habits, the potential confounders by all these factors were also under control. Thirdly, the positive association between hiatal hernia and AEE in our final model corroborates previous reports [4,8,9,21,22] and the validity of statistical analysis. Therefore, we have tentatively concluded that frequent consumption of alcohol and/or tea, independently associates with AEE in Taiwanese asymptomatic men. Based on the magnitude of the odds ratio, alcohol appeared to be a stronger reflux promoter than tea. Moreover, an additive risk of AEE was also observed when drinking both beverages frequently, implicating a role of dietary habits or the subject’s associated lifestyle, in the development of AEE.

AEE is a silent GERD based on the Montreal definition [1], given the specificity of endoscopic mucosal lesions in the diagnosis of EE. Epidemiological studies have shown that the prevalence of AEE among asymptomatic Asian adults undergoing health check, ranged from 12% to 18.5% in Taiwan [6,9,23] and from 1.6% to 7% in other Asian countries [8,21,22,24], respectively. Our data showed a high prevalence of AEE of 21.6% in Taiwanese men, which was consistent with the findings of male predominance in AEE across most Asian large-scale studies [6–9,21,22,24]. In this study, we confined the scope of our investigation of the risk of dietary habits on AEE to the male gender, as men are not only more likely to have EE [25,26], (possibly due to a greater parietal cell mass [27], a higher prevalence of hiatus hernia [28], and poor lifestyle habits such as smoking or alcohol consumption [29,30]), but also report fewer symptoms than women [31]. Thus, men may carry a higher risk of developing severe EE, BE and EAC [26,32]. In addition to the aforementioned hiatal hernia and male gender, data regarding other risk factors of AEE in community-based studies such as obesity, age, and *H*. *pylori* are scant and still inconsistent. For example, unlike the positive association with obesity in EE, data on AEE were mixed as three studies showed a positive association [4,6,22], while 4 other studies, including ours, found no association with obesity [8,9,21]. Moreover, the association between obesity and EE was mainly found in women but not in men [33–35], which may be related to the reflux-promoting effect of estrogen [36]. In addition, older age was not a risk factor for AEE in most studies, including ours, that used asymptomatic non-AEE subjects as controls [6,8,9,21,22,24], except one study that compared subjects with symptomatic EE (SEE) [8]. Despite the association between the severe form of EE (regardless of symptoms) and older age [37], the results on the association between the prevalence of EE and older age in large observational studies were also mixed [25,26,38–40], indicating the possibility that other factors such as hiatus hernia or obesity, which were commonly found in the elderly, may contribute to the severe form of EE [28]. The inverse association with *H*. *pylori* found in two AEE studies [9,21], was consistent with that of other EE studies in Asia [41], though *H*. *pylori* was not investigated in this study. Finally, when compared between patients with AEE and SEE, the risk factors including age, gender, and obesity were inconsistent amongst studies [7,8,21–24], except that psychological distress, overlap of dyspepsia or irritable bowel syndrome were less common in AEE [22,24], indicating a higher pain threshold in patients with AEE.

The lifestyle, particularly, the role of dietary habits on AEE has rarely been studied, and the results from the limited data seemed mixed [6,8,9,21]. Given the assumption of there not being any specific diet restrictions in patients with AEE, this may be an ideal model in which to investigate the relationship between reflux and diets in population-based studies.

Alcohol may promote reflux by reducing LES tone, increasing intra-gastric acidity, decreasing salivary bicarbonate, and also impairing the mucosal barrier in physiological studies regardless of the type and amounts involved [42]. However, large observational studies investigating the effects of alcohol exposure on GERD have been inconsistent, presumably due to different definitions of alcohol consumption, different disease outcome measures, and different population studied, particularly ethnically or culturally-related geographic variations, amongst the studies [13]. To support the reverse causality regarding the outcome measure, we searched available literature and found that 16 out of 22 studies (73%) using EE (or AEE) as an (objective) outcome measure demonstrated an association with alcohol consumption in univariate analysis, as compared to only 4 of 13 studies (31%) using reflux symptoms (or non-erosive reflux disease) as a (subjective) outcome measure (S1 Table), indicating that the concomitant occurrence of reflux symptoms could influence the level of alcohol exposure [43]. In addition, the association between alcohol consumption and GERD (either EE or reflux symptoms) seemed to be more common in Asian studies, as compared to the western counterparts (88% vs. 48%, S1 Table), raising the possibility of contributory roles of genetic predisposition, and culturally-related drinking habits. In fact, acetaldehyde dehydrogenase deficiency, a genetic aberrant predisposition of alcohol metabolism commonly found in Asian populations has recently been linked to BE [44] and esophageal cancer [45] in Asian drinkers. Besides, alcohol drinking is also a culturally-related behavior [30], as Chinese culture encourages binge drinking for middle-aged men during mealtime [46], a scenario resulting in promotion of reflux in this susceptible group, In contrast, heavy episodic drinking can be prevalent in youths in West regardless of whether meals are being eaten [47]. Thus, it is conceivable that frequent alcohol consumption or its associated lifestyle contribute to the occurrence of EE or AEE, particularly in Asian populations.

Tea, processed from the leaf of *Camellia sinensis*, is consumed by two-thirds of the people in the world as a healthy beverage [48]. However, tea is also associated with heartburn [49]. The proposed mechanisms include increased gastric acid secretion through suppressing the *H*. *pylori* proliferation [50], and the decreased LES pressure [51], thereby increasing esophageal acid reflux [52]. Theophylline, a major component in tea, may relax the LES [53] and also has a significant inhibitory effect on visceral pain [54], thus may theoretically contribute to AEE. Recent data have shown that long-term use of LES relaxing medications, such as theophylline, is associated with EAC [55]. However, the epidemiological studies investigating the effects of tea on GERD are scant and the results are conflicting [6,15,29,56–61], possibly because of difficulty in assessing the quantity and the quality of tea in a population-based study. Many culturally-related drinking habits, such as the consumption of black tea, green tea and strong tea [56], along with drinking tea at hot temperatures [62], and adding nutrients such as cream or sugar [49], may contribute differently to the reflux or irritation of esophageal mucosa. For example, drinking strong tea was found highly correlated to the occurrence of GERD in the Han Chinese from a population-based study in Urumqi, China [63]. In our study, 39.2% of the participants were daily tea drinkers. Given the worldwide popularity of tea drinking, clinicians should recognize that even small increases in risk may possibly translate into a larger number of EE cases when the beverage is consumed in large amounts. Future research will be needed to clarify the quantity and the quality of tea consumption, when analyzing the occurrence of GERD in heavy tea drinkers.

Coffee is also a potent stimulant of gastric acid secretion and has similarly been associated with heartburn [64,65]. However, the results of the effects of coffee on LES pressure and postprandial acid reflux time, or the number of acid reflux episodes, were conflicting [66–69]. A recent meta-analysis of epidemiological studies showed no association between coffee consumption and GERD [70]. Other diets which included chocolate, tomato/citric juices, sweet food, and spicy food have also been found to be associated with acid regurgitation or heartburn; however, none have been reported with EE [6,13,56].

There have been limitations in our study. First, because the sample examined in this study was taken from a population of self-selection for those undergoing a health check-up, the result of seeing a prevalent rate of AEE may not be generalized to fit the overall population in Taiwan. However, such self-paid, health check-ups are generally affordable and easily accessible to most residents in Taiwan, who care to know their own health status, particularly for those interested in early cancer screening. As we had already excluded 417 subjects experiencing reflux symptoms, and those taking medications or drugs that potentially impair esophageal sphincter function or cause mucosal injury, all of whom were found to have a prevalence of 35.5% for esophagitis or even more severe lesions (Table 1), those who were included in our study would appear to represent apparently healthy Taiwanese men to some extent. Secondly, because it is difficult to accurately assess the exposure of beverage consumption without objective biomarkers in epidemiological studies [71], we assumed that the frequency of alcohol or tea consumption for a duration of at least 3 months represented the dietary habit or life-style habit related to that beverage and paralleled the amount of intake. Further studies are needed to investigate the concentrations, fermentation methods, accompanying food or additive nutrients, and drinking temperatures of both types of beverages, and if possible, to employ objective biomarkers to validate the true exposure. However, because the data of dietary habits and symptoms were collected immediately before the endoscopy, kept blind to the endoscopists, and a photo confirmation study was reviewed by a different experienced endoscopist, we were able to minimize or avoid information bias and misclassification. Thirdly, it is also possible that food items or lifestyles such as smoking or large meals that accompanies alcohol or tea consumption confound the occurrence of AEE, and were not recorded in our study. However, the possibility of such biased conclusion is unlikely to be fully explained by a clear dose-response relationship existing between AEE and the frequency of alcohol or tea consumption. Further studies are needed to clarify this issue.

## Conclusion

In conclusion, our present study demonstrated that frequent consumption of alcohol and tea increased the risk of AEE in Taiwanese men. Future research to corroborate our hypotheses is warranted for these two common beverages, both of which have been strongly embedded in our dietary culture.

## Supporting information

S1 Table Alcohol consumption and the risk of erosive esophagitis and reflux symptoms from epidemiological studies. (DOC)

## References

[1] Vakil N, van Zanten SV, Kahrilas P, Dent J, Jones R; Global Consensus Group. The Montreal definition and classification of gastroesophageal reflux disease: A global evidence-based consensus. Am J Gastroenterol. 2006;101:1900–1920. 10.1111/j.1572-0241.2006.00630.x 16928254

[2] Goh KL. Gastroesophageal reflux disease in Asia: A historical perspective and present challenges. J Gastroenterol Hepatol. 2011;26 Suppl 1:2–10. 10.1111/j.1440-1746.2010.06534.x 21199509

[3] Dent J, Becher A, Sung J, Zou D, Agréus L, Bazzoli F. Systematic review: Patterns of reflux-induced symptoms and esophageal endoscopic findings in large-scale surveys. Clin Gastroenterol Hepatol. 2012;10:863–873.e3. 10.1016/j.cgh.2012.02.028 22401904

[4] Ronkainen J, Aro P, Storskrubb T, Johansson SE, Lind T, Bolling-Sternevald E, et al High prevalence of gastroesophageal reflux symptoms and esophagitis with or without symptoms in the general adult Swedish population: A Kalixanda study report. Scand J Gastroenterol. 2005;40:275–285. 15932168 10.1080/00365520510011579

[5] Zagari RM, Fuccio L, Wallander MA Johansson S, Fiocca R, Casanova S, et al Gastro-oesophageal reflux symptoms, oesophagitis and Barrett's oesophagus in the general population: the Loiano-Monghidoro study. Gut. 2008;57:1354–1359. 10.1136/gut.2007.145177 18424568

[6] Wang FW, Tu MS, Chuang HY, Yu HC, Cheng LC, Hsu PI. Erosive Esophagitis in Asymptomatic Subjects: Risk Factors. Dig Dis Sci. 2010;55:1320–1324. 10.1007/s10620-009-0888-z 19685186

[7] Nozu T, Komiyama H. Clinical characteristics of asymptomatic esophagitis. J Gastroenterol 2008;43:27–31. 10.1007/s00535-007-2120-2 18297432

[8] Cho JH, Kim HM, Ko GJ, Woo ML, Moon CM, Kim YJ, et al Old age and male sex are associated with increased risk of asymptomatic erosive esophagitis: analysis of data from local health examinations by the Korean National Health Insurance Corporation. J Gastroenterol Hepatol. 2011;26:1034–1038. 10.1111/j.1440-1746.2011.06686.x 21299618

[9] Wang PC, Hsu CS, Tseng TC, Hsieh TC, Chen CH, Su WC, et al Male sex, hiatus hernia, and Helicobacter pylori infection associated with asymptomatic erosive esophagitis. J Gastroenterol Hepatol. 2012;27:586–591. 10.1111/j.1440-1746.2011.06881.x 21871022

[10] Ronkainen J, Talley NJ, Storskrubb T, Johansson SE, Lind T, Vieth M, et al Erosive esophagitis is a risk factor for Barrett's esophagus: a community-based endoscopic follow-up study. Am J Gastroenterol 2011;106:1946–1952. 10.1038/ajg.2011.326 21946284

[11] Gerson LB, Shetler K, Triadafilopoulos G. Prevalence of Barrett's esophagus in asymptomatic individuals. Gastroenterology. 2002;123:461–467. 12145799 10.1053/gast.2002.34748

[12] Lagergren J, Bergström R, Lindgren A, Nyrén O. Symptomatic gastroesophageal reflux as a risk factor for esophageal adenocarcinoma. N Engl J Med. 1999;340:825–831. 10.1056/NEJM199903183401101 10080844

[13] Kaltenbach T, Crockett S, Gerson LB. Are lifestyle measures effective in patients with gastroesophageal reflux disease? An evidence-based approach. Arch Intern Med. 2006;166:965–971. 10.1001/archinte.166.9.965 16682569

[14] Katz PO, Gerson LB, Vela MF. Guidelines for the diagnosis and management of gastroesophageal reflux disease. Am J Gastroenterol. 2013;108:308–328. 10.1038/ajg.2012.444 23419381

[15] Nilsson M, Johnsen R, Ye W, Hveem K, Lagergren J. Lifestyle related risk factors in the aetiology of gastro-oesophageal reflux. Gut. 2004;53:1730–1735. 10.1136/gut.2004.043265 15542505 PMC1774312

[16] Locke GR 3rd, Talley NJ, Fett SL, Zinsmeister AR, Melton LJ 3rd. Prevalence and clinical spectrum of gastroesophageal reflux: A population-based study in Olmsted County, Minnesota. Gastroenterology. 1997;112:1448–1456. 9136821 10.1016/s0016-5085(97)70025-8

[17] Bytzer P. Information bias in endoscopic assessment. Am J Gastroenterol. 2007;102:1585–1587. 10.1111/j.1572-0241.2006.00911.x 17686062

[18] Lundell LR, Dent J, Bennett JR, Blum AL, Armstrong D, Galmiche JP, et al Endoscopic assessment of oesophagitis: Clinical and functional correlates and further validation of the Los Angeles classification. Gut. 1999;45:172–180. 10403727 10.1136/gut.45.2.172 PMC1727604

[19] Chu NF. Prevalence of obesity in Taiwan. Obes Rev. 2005;6:271–274. 10.1111/j.1467-789X.2005.00175.x 16246212

[20] Mickey RM, Greenland S. The impact of confounder selection criteria on effect estimation. Am J Epidemiol. 1989;129:125–137. 2910056 10.1093/oxfordjournals.aje.a115101

[21] Peng S, Cui Y, Xiao YL, Xiong LS, Hu PJ, Li CJ, et al Prevalence of erosive esophagitis and Barrett's esophagus in the adult Chinese population. Endoscopy. 2009;41:1011–1017. 10.1055/s-0029-1215291 19967617

[22] Choi JY, Jung HK, Song EM, Shim KN, Jung SA. Determinants of symptoms in gastroesophageal reflux disease: Nonerosive reflux disease, symptomatic, and silent erosive reflux disease. Eur J Gastroenterol Hepatol. 2013;25:764–771. 10.1097/MEG.0b013e32835f594c 23459104

[23] Lei WY, Yu HC, Wen SH, Liu TT, Yi CH, Wang CC, et al Predictive factors of silent reflux in subjects with erosive esophagitis. Dig Liver Dis 2015;47:24–29. 10.1016/j.dld.2014.09.017 25308612

[24] Lee D, Lee KJ, Kim KM, Lim SK. Prevalence of asymptomatic erosive esophagitis and factors associated with symptom presentation of erosive esophagitis. Scand J Gastroenterol. 2013;48:906–912. 10.3109/00365521.2013.812236 23834193

[25] Moayyedi P, Talley NJ. Gastro-oesophageal reflux disease. Lancet 2006;367:2086–2100. 10.1016/S0140-6736(06)68932-0 16798392

[26] Labenz J, Jaspersen D, Kulig M, Leodolter A, Lind T, Meyer-Sabellek W, et al Risk factor for erosive esophagitis: A multivariate analysis based on the ProGRED study initiative. Am J Gastroenterol 2004;99:1652–1656. 10.1111/j.1572-0241.2004.30390.x 15330897

[27] Kekki M, Samloff IM, Ihamäki T, Varis K, Siurala M. Age- and sex-related behaviour of gastric acid secretion at the population level. Scand J Gastroenterol 1982;17:737–43. 7188552 10.3109/00365528209181087

[28] Menon S, Trudgill N. Risk factors in the aetiology of hiatus hernia: a meta-analysis. Eur J Gastroenterol Hepatol 2011;23:133–138. 10.1097/MEG.0b013e3283426f57 21178776

[29] Chen TS, Chang FY. The prevalence and risk factors of reflux esophagitis among adult Chinese population in Taiwan. J Clin Gastroenterol. 2007;41:819–822. 10.1097/01.mcg.0000225658.30803.79 17881927

[30] World Health Organization. Global status report on alcohol and health 2014. [Available from: http://www.who.int/substance_abuse/publications/global_alcohol_report/en/]

[31] Chen Z, Thompson SK, Jamieson GG, Devitt PG, Watson DI. Effect of sex on symptoms associated with gastroesophageal reflux. Arch Surg. 2011;146:1164–1169. 10.1001/archsurg.2011.248 22006875

[32] Pohl H, Wrobel K, Bojarski C, Voderholzer W, Sonnenberg A, Rösch T, et al Risk factors in the development of esophageal adenocarcinoma. Am J Gastroenterol 2013;108:200–207. 10.1038/ajg.2012.387 23247577

[33] Nocon M, Labenz J, Jaspersen D, Meyer-Sabellek W, Stolte M, Lind T, et al Association of body mass index with heartburn, regurgitation and esophagitis: results of the Progression Gastroesophageal Reflux Disease Study. J Gastroenterol Hepatol. 2007;22:1728–1731. 10.1111/j.1440-1746.2006.04549.x 17914941

[34] Lien HC, Chang CS, Yeh HZ, Ko CW, Chang HY, Cheng KF, et al Increasing prevalence of erosive esophagitis among Taiwanese aged 40 and above: a comparison between two time periods. J Clin Gastroenterol 2009;43: 926–932. 10.1097/MCG.0b013e318191e9d5 19384245

[35] Nilsson M, Lundegardh G, Carling L, Ye W, Lagergren J. Body mass and reflux oesophagitis: an oestrogen-dependent association? Scand J Gastroenterol. 2002;37:626–630. 12126237 10.1080/00365520212502

[36] Zheng Z, Margolis KL, Liu S, Tinker LF, Ye W; Women's Health Initiative Investigators. Effects of estrogen with and without progestin and obesity on symptomatic gastroesophageal reflux. Gastroenterology 2008;135:72–81. 10.1053/j.gastro.2008.03.039 18502208 PMC2725519

[37] Johnson DA, Fennerty MB. Heartburn severity underestimates erosive esophagitis severity in elderly patients with gastroesophageal reflux disease. Gastroenterology 2004;126:660–664. 14988819 10.1053/j.gastro.2003.12.001

[38] Menon S, Jayasena H, Nightingale P, Trudgill NJ. Influence of age and sex on endoscopic findings of gastrooesophageal reflux disease: an endoscopy database study. Eur J Gastroenterol Hepatol. 2011;23:389–395. 10.1097/MEG.0b013e328345d429 21448069

[39] Minatsuki C, Yamamichi N, Shimamoto T, Kakimoto H, Takahashi Y, Fujishiro M, et al Background factors of reflux esophagitis and non-erosive reflux disease: a cross-sectional study of 10,837 subjects in Japan. PLoS One 2013;8:e69891 10.1371/journal.pone.0069891 23922844 PMC3724738

[40] Kim N, Lee SW, Cho SI, Park CG, Yang CH, Kim HS, et al The prevalence of and risk factors for erosive oesophagitis and non-erosive reflux disease: a nationwide multicentre prospective study in Korea. Aliment Pharmacol Ther 2008;27:173–185. 10.1111/j.1365-2036.2007.03561.x 17973646

[41] Raghunath A, Hungin AP, Wooff D, Childs S. Prevalence of Helicobacter pylori in patients with gastro-oesophageal reflux disease: systematic review. BMJ 2003;326:737 10.1136/bmj.326.7392.737 12676842 PMC152634

[42] Bujanda L. The effects of alcohol consumption upon the gastrointestinal tract. Am J Gastroenterol. 2000;95:3374–3382. 10.1111/j.1572-0241.2000.03347.x 11151864

[43] Shapiro M, Green C, Bautista JM, Dekel R, Risner-Adler S, Whitacre R, et al Assessment of dietary nutrients that influence perception of intra-oesophageal acid reflux events in patients with gastro-oesophageal reflux disease. Aliment Pharmacol Ther. 2007;25:93–101. 10.1111/j.1365-2036.2006.03170.x 17229224

[44] Ren LL, Yan TT, Wang ZH, Bian ZL, Yang F, Hong J, et al Alcohol consumption and the risk of Barrett's esophagus: A comprehensive meta-analysis. Sci Rep. 2015;5:16048 10.1038/srep16048 26542211 PMC4635354

[45] Brooks PJ, Enoch MA, Goldman D, Li TK, Yokoyama A. The alcohol flushing response: An unrecognized risk factor for esophageal cancer from alcohol consumption. PLoS Med. 2009;6:e50 10.1371/journal.pmed.1000050 19320537 PMC2659709

[46] Li Y, Jiang Y, Zhang M, Yin P, Wu F, Zhao W. Drinking behaviour among men and women in China: The 2007 China Chronic Disease and Risk Factor Surveillance. Addiction. 2011;106:1946–1956. 10.1111/j.1360-0443.2011.03514.x 21771141

[47] Nazareth I, Walker C, Ridolfi A, Aluoja A, Bellon J, Geerlings M, et al Heavy episodic drinking in Europe: A cross section study in primary care in six European countries. Alcohol Alcohol. 2011;46:600–606. 10.1093/alcalc/agr078 21733834

[48] Khan N, Mukhtar H. Tea and health: Studies in humans. Curr Pharm Des. 2013;19:6141–6147. 23448443 10.2174/1381612811319340008 PMC4055352

[49] Feldman M, Barnett C. Relationships between the acidity and osmolality of popular beverages and reported postprandial heartburn. Gastroenterology. 1995;108:125–131. 7806034 10.1016/0016-5085(95)90016-0

[50] Takabayashi F, Harada N, Yamada M, Murohisa B, Oguni I. Inhibitory effect of green tea catechins in combination with sucralfate on Helicobacter pylori infection in Mongolian gerbils. J Gastroenterol. 2004;39:61–63. 10.1007/s00535-003-1246-0 14767736

[51] Gudjonsson H, McAuliffe TL, Kaye MD. The effect of coffee and tea upon lower esophageal sphincteric function. Laeknabladid. 1995;81:484–488. 20065484

[52] Ko CW, Chang CS, Kao CH, Chen GH, Yen CY. Effect of green tea on esophageal transit and gastric emptying in patients with heartburn. Gastroenterol J Taiwan. 2002:19:116–123.

[53] Berquist WE, Rachelefsky GS, Kadden M, Siegel SC, Katz RM, Mickey MR, et al Effect of theophylline on gastroesophageal reflux in normal adults. J Allergy Clin Immunol. 1981;67:407–411. 7229228 10.1016/0091-6749(81)90087-7

[54] Rao SS, Mudipalli RS, Mujica V, Utech CL, Zhao X, Conklin JL. An open-label trial of theophylline for functional chest pain. Dig Dis Sci. 2002;47:2763–2768. 12498299 10.1023/a:1021017524660

[55] Alexandre L, Broughton T, Loke Y, Beales IL. Meta-analysis: risk of esophageal adenocarcinoma with medications which relax the lower esophageal sphincter. Dis Esophagus. 2012;25:535–544. 10.1111/j.1442-2050.2011.01285.x 22129441

[56] Du J, Liu J, Zhang H, Yu CH, Li YM. Risk factors for gastroesophageal reflux disease, reflux esophagitis and non-erosive reflux disease among Chinese patients undergoing upper gastrointestinal endoscopic examination. World J Gastroenterol. 2007;13:6009–6015. 10.3748/wjg.v13.45.6009 18023091 PMC4250882

[57] Murao T, Sakurai K, Mihara S, Marubayashi T, Murakami Y, Sasaki Y. Lifestyle change influences on GERD in Japan: A study of participants in a health examination program. Dig Dis Sci. 2011;56:2857–2864. 10.1007/s10620-011-1679-x 21487772 PMC3179841

[58] Kubo A, Block G, Quesenberry CP Jr, Buffler P, Corley DA. Dietary guideline adherence for gastroesophageal reflux disease. BMC Gastroenterol. 2014;14:144 10.1186/1471-230X-14-144 25125219 PMC4139138

[59] Chih PC, Yang YC, Wu JS, Chang YF, Lu FH, Chang CJ. Overweight associated with increased risk of erosive esophagitis in a non-obese Taiwanese population. PLoS One. 2013;8:e77932 10.1371/journal.pone.0077932 24223746 PMC3815304

[60] Nocon M, Labenz J, Willich SN. Lifestyle factors and symptoms of gastro-oesophageal reflux—a population-based study. Aliment Pharmacol Ther. 2006;23:169–174. 10.1111/j.1365-2036.2006.02727.x 16393294

[61] Ou JL, Tu CC, Hsu PI, Pan MH, Lee CC, Tsay FW, et al Prevalence and risk factors of erosive esophagitis in Taiwan. J Chin Med Assoc. 2012;75:60–64. 10.1016/j.jcma.2011.12.008 22340738

[62] Chen Z, Chen Q, Xia H, Lin J. Green tea drinking habits and esophageal cancer in southern China: A case-control study. Asian Pac J Cancer Prev. 2011;12:229–233. 21517263

[63] Niu CY, Zhou YL, Yan R, Mu NL, Gao BH, Wu FX, et al Incidence of gastroesophageal reflux disease in Uygur and Han Chinese adults in Urumqi. World J Gastroenterol. 2012;18:7333–7340. 10.3748/wjg.v18.i48.7333 23326142 PMC3544039

[64] Cohen S. Pathogenesis of coffee-induced gastrointestinal symptoms. N Engl J Med. 1980;303:122–124. 10.1056/NEJM198007173030302 7383069

[65] Cohen S, Booth GH Jr. Gastric acid secretion and lower-esophageal-sphincter pressure in response to coffee and caffeine. N Engl J Med. 1975;293:897–899. 10.1056/NEJM197510302931803 1177987

[66] Thomas FB, Steinbaugh JT, Fromkes JJ, Mekhjian HS, Caldwell JH. Inhibitory effect of coffee on lower esophageal sphincter pressure. Gastroenterology. 1980;79:1262–1266. 7002705

[67] Pehl C, Pfeiffer A, Wendl B, Kaess H. The effect of decaffeination of coffee on gastro-oesophageal reflux in patients with reflux disease. Aliment Pharmacol Ther. 1997;11:483–486. 9218070 10.1046/j.1365-2036.1997.00161.x

[68] Wendl B, Pfeiffer A, Pehl C, Schmidt T, Kaess H. Effect of decaffeination of coffee or tea on gastro-oesophageal reflux. Aliment Pharmacol Ther. 1994;8:283–287. 7918922 10.1111/j.1365-2036.1994.tb00289.x

[69] Boekema PJ, Samsom M, Smout AJ. Effect of coffee on gastro-oesophageal reflux in patients with reflux disease and healthy controls. Eur J Gastroenterol Hepatol. 1999;11:1271–1276. 10563539 10.1097/00042737-199911000-00015

[70] Kim J, Oh SW, Myung SK, Kwon H, Lee C, Yun JM, et al Association between coffee intake and gastroesophageal reflux disease: A meta-analysis. Dis Esophagus. 2014;27:311–317. 10.1111/dote.12099 23795898

[71] Kipnis V, Subar AF, Midthune D, Freedman LS, Ballard-Barbash R, Troiano RP, et al Structure of dietary measurement error: results of the OPEN biomarker study. Am J Epidemiol 2003;158:14–21; discussion 22–6. 12835281 10.1093/aje/kwg091

