# Supplementary material for: Alcohol and tea consumption are associated with asymptomatic erosive esophagitis in Taiwanese men
Source: PLoS One. 2017 Mar 6;12(3):e0173230. doi: 10.1371/journal.pone.0173230 (PMC5338804; doi:10.1371/journal.pone.0173230)
Supplement: S1 Table — (DOC) [file pone.0173230.s001.doc]

| Supplementary table 1. Alcohol consumption and the risk of erosive esophagitis and reflux symptoms from epidemiological studies | | | | | | |
| --- | --- | --- | --- | --- | --- | --- |
| Study | Country | No. of patients | Outcome* | Definition of drinker | Univariate | Multivariate |
| Murao , 2011 [1] | Japan (E) | 2853 | AEE | Drinking alcohol often or everyday | Significant | NS |
| Peng , 2009 [2] | China (E) | 2580 | AEE | NA | Significant | aOR 3.17 (1.44-6.97) |
| Wang , 2010 [3] | Taiwan (E) | 572 | AEE | >3 drinks/week, | Significant | NS |
| Wang , 2012 [4] | Taiwan (E) | 594 | AEE | >140g/week | NS | NS |
| Cho , 2011 [5] | Korea (E) | 5301 | AEE | NA | NS | NS |
| Akiyama, 2008 [6] | Japan (E) | 463 | EE | heavy drinkers (more than 50g per day) | Significant | NA |
| Chen , 2007 [7] | Taiwan (E) | 482 | EE | >3 drinks/week, | Significant | NS |
| Chih , 2013 [8] | Taiwan (E) | 7352 | EE | >1 drink/week for >6 months | Significant | aOR 1.31 (1.12-1.54) |
| Cho , 2011 [5] | Korea (E) | 5301 | EE | NA | NS | NS |
| Gunji , 2011 [9] | Japan (E) | 9840 | EE | >280g/week | Significant | aOR 1.276 (1.085-1.501) |
| Kim , 2008 [10] | Korea (E) | 25536 | EE | >once/week | Significant | aOR 1.5 (1.21-1.81) |
| Kim , 2011 [11] | Korea (E) | 7278 | EE | >80g/day | Significant | aOR 1.34 (1.04-1.72) |
| Lee , 2001 [12] | Korea (E) | 7015 | EE | >3 drinks/week, each >35g | Significant | NS |
| Matsuzaki, 2015 [13] | Japan (E) | 2608 | EE | ≥1 days / week | NS | NS |
| Minatsuki, 2013 [14] | Japan (E) | 10837 | EE | drink sometimes, drink almost everyday, or drink everyday | Significant | 1.17 (1.07–1.29) |
| Ou , 2012 [15] | Taiwan (E) | 2040 | EE | >3 drinks/week, | Significant | NS |
| Rosaida , 2004 [16] | Malaysia (E) | 1000 | EE | NA | Significant | aOR 3.22 (1.26-8.22) |
| Avidan , 2001 [17] | USA (W) | 4961 | EE | Alcohol use within 12 months | Significant | NS |
| Avidan , 2001 [18] | USA (W) | 1197 | EE | NA | Significant | aOR 1.47 (1.08-1.99) |
| El-Serag , 2005 [19] | USA (W) | 164 | EE | g/day | NS | NS |
| Stene-Larsen , 1988 [20] | Norway (W) | 1224 | EE | Alcohol abuse | Significant | NA |
| Kang , 1999 [21] | UK and Singapore (B) | 385 | EE | Number of units multiplying years | NS | NS |
| Kim , 2008 [10] | Korea (E) | 25536 | NERD | >once/week | NS | NS |
| Minatsuki, 2013 [14] | Japan (E) | 10837 | NERD | drink sometimes, drink almost everyday, or drink everyday | Significant | 1.08 (1.02–1.14) |
| Rosaida , 2004 [16] | Malaysia (E) | 1000 | NERD | NA | NS | NS |
| Wang , 2004 [22] | China (E) | 2789 | reflux symptoms | >210 g/week | Significant | aOR 2.85 (1.67-4.49) |
| El-Serag , 2005 [19] | USA (W) | 371 | reflux symptoms | g/day | NS | NS |
| Haque , 2000 [23] | New Zealand (W) | 778 | reflux symptoms | Drink number/week in the last 1 y | NS | NS |
| Lagergren , 2000 [24] | Sweden (W) | 820 | reflux symptoms | >70 g/week | NS | NS |
| Locke , 1999 [25] | USA (W) | 1524 | reflux symptoms | >7 drinks/week | Significant | aOR 1.9 (1.1-3.3) |
| Mohammed I, 2003 [26] | UK (W) | 4480 | reflux symptoms | >28 units per week for a man and >21 units per week for a woman | NS | NS |
| Mohammed I, 2005 [27] | UK (W) | 4000 | reflux symptoms | >30 units/week for men and  >20 units/week for women | Significant | aOR: 2.96 (1.45–6.06) |
| Nilsson , 2004 [28] | Norway (W) | 43363 | reflux symptoms | No. of drinks within 2 weeks at baseline | NS | NS |
| Nocon , 2006 [29] | Germany (W) | 7124 | reflux symptoms | ≥1 drink/day | NS except wine and spirits wine | aOR 1.63 (1.12-2.36) |
| Ruhl , 1999 [30] | USA (W) | 12349 | hospitalization reflux disease | >2 drinks/day at baseline | NS | NS |
| Ruigómez A, 2004 [31] | UK (W) | 1996 | reflux symptoms | units per week | NS | NS |
| Zheng , 2007 [32] | Sweden (W) | 25466 | reflux symptom | g/month | NS | NS |
| Stanghellini , 1999 [33] | Canada, USA, Switzerland, Netherlands, Italy, Japan, Denmark, Finland, Sweden and Norway. (B) | 5581 | reflux symptoms | Daily or weekly alcohol consumption | NS | NS |
| * Disease outcome compared with healthy population.  E, eastern country; W, western country; B, both eastern and western countries.  No., number; AEE, asymptomatic erosive esophagitis; EE, erosive esophagitis; NERD, non-erosive reflux disease; NA, not applicable; NS, non-significant, aOR, adjusted odds ratio. | | | | | | |

**References:**

1. Murao T, Sakurai K, Mihara S, Marubayashi T, Murakami Y, Sasaki Y. Lifestyle change influences on GERD in Japan: A study of participants in a health examination program. *Dig Dis Sci.* 2011;56:2857-2864. DOI: 10.1007/s10620-011-1679-x. PMID: 21487772.
2. Peng S, Cui Y, Xiao YL, Xiong LS, Hu PJ, Li CJ, et al. Prevalence of erosive esophagitis and Barrett's esophagus in the adult Chinese population. *Endoscopy.* 2009;41:1011-1017. DOI: 10.1055/s-0029-1215291. PMID: 19967617.
3. Wang FW, Tu MS, Chuang HY, Yu HC, Cheng LC, Hsu PI. Erosive esophagitis in asymptomatic subjects: risk factors. *Dig Dis Sci.* 2010;55:1320-1324. DOI: 10.1007/s10620-009-0888-z. PMID: 19685186.
4. Wang PC, Hsu CS, Tseng TC, Hsieh TC, Chen CH, Su WC, et al. Male sex, hiatus hernia, and Helicobacter pylori infection associated with asymptomatic erosive esophagitis. *J Gastroenterol Hepatol.* 2012;27:586-591. DOI: 10.1111/j.1440-1746.2011.06881.x. PMID: 21871022.
5. Cho JH, Kim HM, Ko GJ, Woo ML, Moon CM, Kim YJ, et al. Old age and male sex are associated with increased risk of asymptomatic erosive esophagitis: Analysis of data from local health examinations by the Korean National Health Insurance Corporation. *J Gastroenterol Hepatol.* 2011;26:1034-1038. DOI: 10.1111/j.1440-1746.2011.06686.x. PMID: 21299618.
6. Akiyama T, Inamori M, Iida H, Mawatari H, Endo H, Hosono K, et al. Alcohol consumption is associated with an increased risk of erosive esophagitis and Barrett's epithelium in Japanese men. *BMC Gastroenterol.* 2008;8:58. DOI: 10.1186/1471-230X-8-58. PMID: 19077221.
7. Chen TS, Chang FY. The prevalence and risk factors of reflux esophagitis among adult Chinese population in Taiwan. *J Clin Gastroenterol.* 2007;41:819-822. PMID: 17881927.
8. Chih PC, Yang YC, Wu JS, Chang YF, Lu FH, Chang CJ. Overweight associated with increased risk of erosive esophagitis in a non-obese Taiwanese population. *PLoS One.* 2013;8:e77932. DOI: 10.1371/journal.pone.0077932. PMID: 24223746.
9. Gunji T, Sato H, Iijima K, Fujibayashi K, Okumura M, Sasabe N, et al. Risk factors for erosive esophagitis: A cross-sectional study of a large number of Japanese males. *J Gastroenterol.* 2011;46:448-455. DOI: 10.1007/s00535-010-0359-5. PMID: 21229366.
10. Kim BJ, Cheon WS, Oh HC, Kim JW, Park JD, Kim JG. The prevalence of and risk factors for erosive oesophagitis and non-erosive reflux disease: A nationwide multicentre prospective study in Korea. *Aliment Pharmacol Ther.* 2008;27:173-185. PMID: 17973646
11. Kim BJ, Cheon WS, Oh HC, Kim JW, Park JD, Kim JG.. Prevalence and risk factor of erosive esophagitis observed in Korean National Cancer Screening Program. *J Korean Med Sci.* 2011;26:642-646. DOI: 10.3346/jkms.2011.26.5.642. PMID: 21532855.
12. Lee SJ, Song CW, Jeen YT, Chun HJ, Lee HS, Um SH, et al. Prevalence of endoscopic reflux esophagitis among Koreans. *J Gastroenterol Hepatol.* 2001;16:373-376. PMID: 11354273.
13. Matsuzaki J, Suzuki H, Kobayakawa M, Inadomi JM, Takayama M, Makino K, et al. Association of Visceral Fat Area, Smoking, and Alcohol Consumption with Reflux Esophagitis and Barrett's Esophagus in Japan. *PLoS One.* 2015;10:e0133865. DOI: 10.1371/journal.pone.0133865. PMID: 26225858.
14. Minatsuki C, Yamamichi N, Shimamoto T, Kakimoto H, Takahashi Y, Fujishiro M, et al. Background factors of reflux esophagitis and non-erosive reflux disease: A cross-sectional study of 10,837 subjects in Japan. *PLoS One.* 2013;8:e69891. DOI: 10.1371/journal.pone.0069891. PMID: 23922844.
15. Ou JL, Tu CC, Hsu PI, Pan MH, Lee CC, Tsay FW, et al. Prevalence and risk factors of erosive esophagitis in Taiwan. *J Chin Med Assoc.* 2012;75:60-64. DOI: 10.1016/j.jcma.2011.12.008. PMID: 22340738.
16. Rosaida MS, Goh KL. Gastro-oesophageal reflux disease, reflux oesophagitis and non-erosive reflux disease in a multiracial Asian population: A prospective, endoscopy based study. *Eur J Gastroenterol Hepatol.* 2004;16:495-501. PMID: 15097043.
17. Avidan B, Sonnenberg A, Schnell TG, Sontag SJ. Risk factors for erosive reflux esophagitis: A case-control study. *Am J Gastroenterol.* 2001;96:41-46. PMID: 11197285.
18. Avidan B, Sonnenberg A, Schnell TG, Sontag SJ. No association between gallstones and gastroesophageal reflux disease. *Am J Gastroenterol.* 2001;96:2858-2862. PMID: 11693317.
19. El-Serag HB, Satia JA, Rabeneck L. Dietary intake and the risk of gastro-oesophageal reflux disease: A cross sectional study in volunteers. *Gut.* 2005;54:11-17. PMID: 15591498.
20. Stene-Larsen G, Weberg R, Frøyshov Larsen I, Bjørtuft O, Hoel B, Berstad A. Relationship of overweight to hiatus hernia and reflux oesophagitis. *Scand J Gastroenterol.* 1988;23:427-432. PMID: 3381064.
21. Kang JY, Ho KY. Different prevalences of reflux oesophagitis and hiatus hernia among dyspeptic patients in England and Singapore. *Eur J Gastroenterol Hepatol.* 1999;11:845-850. PMID: 10514115.
22. Wang JH, Luo JY, Dong L, Gong J, Tong M. Epidemiology of gastroesophageal reflux disease: A general population-based study in Xi'an of Northwest China. *World J Gastroenterol.* 2004;10:1647-1651. PMID: 15162542.
23. Haque M, Wyeth JW, Stace NH, Talley NJ, Green R. Prevalence, severity and associated features of gastro-oesophageal reflux and dyspepsia: A population-based study. *N Z Med J.* 2000;113:178-81. PMID: 10917077.
24. Lagergren J, Bergström R, Nyrén O. No relation between body mass and gastro-oesophageal reflux symptoms in a Swedish population based study. *Gut.* 2000;47:26-29. PMID: 10861260.
25. Mohammed I, Cherkas LF, Riley SA, Spector TD, Trudgill NJ. Genetic influences in gastro-oesophageal reflux disease: A twin study. *Gut.* 2003;52:1085-1089. PMID: 12865263.
26. Mohammed I, Nightingale P, Trudgill NJ. Risk factors for gastro-oesophageal reflux disease symptoms: A community study. *Aliment Pharmacol Ther.* 2005;21:821-827. PMID: 15801917.
27. Locke GR 3rd, Talley NJ, Fett SL, Zinsmeister AR, Melton LJ 3rd. Risk factors associated with symptoms of gastroesophageal reflux. *Am J Med.* 1999;106:642-649. PMID: 10378622.
28. Nilsson M, Johnsen R, Ye W, Hveem K, Lagergren J. Lifestyle related risk factors in the aetiology of gastro-oesophageal reflux. *Gut.* 2004;53:1730-1735. PMID: 15542505.
29. Nocon M, Labenz J, Willich SN. Lifestyle factors and symptoms of gastro-oesophageal reflux -- a population-based study. *Aliment Pharmacol Ther.* 2006;23:169-174. PMID: 16393294.
30. Ruhl CE, Everhart JE. Overweight, but not high dietary fat intake, increases risk of gastroesophageal reflux disease hospitalization: The NHANES I Epidemiologic Follow up Study. First National Health and Nutrition Examination Survey. *Ann Epidemiol.* 1999;9:424-435. PMID: 10501410.
31. Ruigómez A, García Rodríguez LA, Wallander MA, Johansson S, Graffner H, Dent J. Natural history of gastro-oesophageal reflux disease diagnosed in general practice. *Aliment Pharmacol Ther.* 2004;20:751-760. PMID: 15379835.
32. Zheng Z, Nordenstedt H, Pedersen NL, Lagergren J, Ye W. Lifestyle factors and risk for symptomatic gastroesophageal reflux in monozygotic twins. *Gastroenterology.* 2007;132:87-95. PMID: 17241862.
33. Stanghellini V. Relationship between upper gastrointestinal symptoms and lifestyle, psychosocial factors and comorbidity in the general population: Results from the Domestic/International Gastroenterology Surveillance Study (DIGEST). *Scand J Gastroenterol* *Suppl.* 1999;231:29-37. PMID: 10565621.
